# Doxorubicin-Induced Cardiotoxicity and the Emerging Role of SGLT2 Inhibitors: From Glycemic Control to Cardio-Oncology

**DOI:** 10.3390/ph18050681

**Published:** 2025-05-03

**Authors:** Iacob-Daniel Goje, Greta-Ionela Goje, Valentin Laurențiu Ordodi, Valentina Gabriela Ciobotaru, Vlad Sabin Ivan, Roxana Buzaș, Oana Tunea, Florina Bojin, Daniel-Florin Lighezan

**Affiliations:** 1 Department of Medical Semiology I, “Victor Babes” University of Medicine and Pharmacy, No. 2 Eftimie Murgu Square, 300041 Timisoara, Romania; daniel.goje@umft.ro (I.-D.G.); vaciobotaru@gmail.com (V.G.C.); ivan.vlad@umft.ro (V.S.I.); buzas.dana@umft.ro (R.B.); ancusa.oana@umft.ro (O.T.); dlighezan@umft.ro (D.-F.L.); 2 Advanced Cardiology and Hemostaseology Research Center, “Victor Babes” University of Medicine and Pharmacy, No. 2 Eftimie Murgu Square, 300041 Timisoara, Romania; 3 Department I of Nursing, University Clinic of Clinical Skills, “Victor Babes” University of Medicine and Pharmacy, No. 2 Eftimie Murgu Square, 300041 Timisoara, Romania; 4 Faculty of Industrial Chemistry and Environmental Engineering, “Politehnica” University Timisoara, No. 2 Victoriei Square, 300006 Timisoara, Romania; valentin.ordodi@upt.ro; 5 Center for Gene and Cellular Therapies in the Treatment of Cancer Timisoara-OncoGen, Clinical Emergency County Hospital “Pius Brinzeu” Timisoara, No. 156 Liviu Rebreanu, 300723 Timisoara, Romania; florinabojin@umft.ro; 6 Department of Functional Sciences, Immuno-Physiology and Biotechnologies Center, “Victor Babes” University of Medicine and Pharmacy, No. 2 Eftimie Murgu Square, 300041 Timisoara, Romania

**Keywords:** SGLT2 inhibitors, cardiovascular protection, metabolic benefits, cardio-oncology, heart failure, cardiotoxicity, anthracyclines

## Abstract

Cancer remains the second leading cause of death worldwide. Doxorubicin (DOX) is a cornerstone of hematologic malignancy treatment, but it is limited by its dose-dependent cardiotoxicity, leading to systolic and diastolic cardiac dysfunction and, ultimately, dilated hypokinetic cardiomyopathy. Cardio-oncology has emerged as a subspecialty addressing cardiovascular complications in cancer patients, highlighting preventive and therapeutic strategies to reduce cancer therapy-related cardiac dysfunction (CTRCD). Current approaches, including beta-blockers, renin–angiotensin system (RAS) inhibitors, and statins, offer partial cardioprotection. Sodium-glucose cotransporter-2 (SGLT2) inhibitors, initially developed for type 2 diabetes mellitus (T2DM), demonstrate pleiotropic cardioprotective effects beyond glycemic control, including reduced oxidative stress, inflammation, and myocardial remodeling. This review explores the interplay between anthracycline therapy, particularly DOX, and cardiotoxicity while evaluating SGLT2 inhibitors as novel agents in cardio-oncology. Preclinical studies suggest SGLT2 inhibitors attenuate CTRCD by preserving mitochondrial function and inhibiting apoptosis, while clinical trials highlight their efficacy in reducing heart failure (HF) hospitalizations and cardiovascular (CV) mortality. Integrating SGLT2 inhibitors into cardio-oncology protocols could revolutionize the management of CTRCD, enhancing patient outcomes in oncology and cardiovascular care. Considering the emerging evidence, SGLT2 inhibitors may provide significant benefits to patients undergoing anthracycline therapy, particularly those with elevated cardiovascular risk profiles. We recommend that future prospective, large-scale clinical trials further evaluate the efficacy and safety of these agents as cardioprotective therapy to optimize individualized treatment strategies.

## 1. Introduction

Cancer is a diverse and complex illness characterized by genetic and molecular abnormalities that lead to uncontrolled cellular growth and proliferation [1]. Many anti-cancer pharmaceutical agents have been developed and can inhibit tumor proliferation through various mechanisms. Certain medications and chemicals target essential biological enzymes, while others may modify cellular metabolism [2]. Chemotherapy, which employs drugs to eradicate malignant cells, originated following observations of mustard gas’s lethal effects on lymphatic tissues and bone marrow [3].

Since the late 1960s, anthracyclines have been recognized as a fundamental component in treating non-Hodgkin’s lymphoma (NHL), particularly for aggressive forms of the illness. Doxorubicin (DOX), the initial antitumor antibiotic, was introduced, and subsequent clinical trials established it as the most efficacious monotherapy for the treatment of NHL [4]. The combination therapy of cyclophosphamide (C), hydroxydaunorubicin (doxorubicin) (H), oncovin (vincristine) (O), and prednisone (P)—CHOP has long been considered the standard regimen to treat aggressive NHL [5]. Using rituximab, a chimeric monoclonal antibody targeting the CD20 B-cell antigen, in CHOP (R-CHOP) has been shown to enhance the prognosis of patients with diffuse large B-cell lymphoma (DLBCL) [6].

DOX is a key component in hematological chemotherapy protocols. However, its use is sometimes limited by significant cardiotoxicity [4,7]. This adverse effect is dose-dependent and mostly irreversible and, in advanced cases, may progress to dilated hypokinetic cardiomyopathy [7]. Anthracycline-induced cardiotoxicity (AIC) is classified into three distinct categories. The acute form occurs after a single dose, with symptoms emerging within 14 days post-treatment, and is generally reversible. Early-onset chronic cardiotoxicity, the most common presentation, manifests within one year and is characterized by dilated cardiomyopathy. Late-onset chronic cardiotoxicity may develop years or even decades after treatment cessation [8]. New research questions this old classification. It suggests that anthracycline-induced cardiotoxicity may be an ongoing process that starts at the level of myocardial cells and then leads to a gradual loss of function, ultimately resulting in heart failure (HF) [9].

Cardio-oncology is an emerging interdisciplinary field that helps healthcare professionals with the cardiovascular complications associated with cancer, providing care before, during, and after chemotherapy treatment [10]. This dual burden of cancer and chemotherapy toxicity on the heart has created an urgent need for novel cardioprotective strategies that can mitigate the adverse effects of cancer therapies without compromising their efficacy [11]. Current cardioprotective agents, such as beta-blockers, angiotensin-converting enzyme inhibitors (ACE-I)/angiotensin receptor blockers (ARB), and statins, have shown some promise. The 2022 ESC guidelines on cardio-oncology advise using these medications for primary prevention of cancer therapy-related cardiovascular toxicity in high- and very high-risk patients receiving anthracyclines (Class IIa recommendation) [10]. Still, their effectiveness remains limited, highlighting the need for further research and development. This unresolved problem has driven the search for innovative therapies, with sodium-glucose cotransporter-2 (SGLT2) inhibitors emerging as a potential game-changer [12].

Initially developed as glucose-lowering agents for treating type 2 diabetes mellitus (T2DM), SGLT2 inhibitors have demonstrated a broad spectrum of benefits beyond glycemic control. These drugs promote glycosuria and improve metabolic parameters by blocking glucose reabsorption in the renal proximal tubules [13]. The effect of SGLT2 inhibitors extends far beyond the kidneys, providing cardiovascular protective effects and becoming a novel therapeutic option for patients regardless of diabetes status. The pleiotropic effects of SGLT2 inhibitors have sparked interest in their potential applications in preventing and managing cancer therapy-induced cardiotoxicity [14].

SGLT2 inhibitors have demonstrated significant cardioprotective effects, with a class I indication in treating heart failure (HF), regardless of ejection fraction [15]. Milestone clinical trials, such as DAPA-HF and EMPEROR-Reduced, have highlighted the benefits of these agents in reducing hospitalizations for heart failure and improving the survival of HF patients [16,17]. SGLT2 inhibitors’ mechanisms of action include improving endothelial function, reducing oxidative stress and inflammation, and having diuretic effects that contribute to lowering blood pressure. These drugs facilitate the excretion of glucose and sodium, reducing plasma volume and, therefore, cardiac preload and afterload. SGLT2 inhibitors also influence myocardial energy metabolism by increasing the utilization of ketone bodies as a source of energy, which improves mitochondrial function [18].

Preclinical studies suggest that SGLT2 inhibitors may reduce DOX-associated cardiac damage by improving myocardial energy metabolism and reducing oxidative stress [19]. The transition from preclinical to clinical trials confirmed that SGLT2 inhibitors improve clinical outcomes among cancer patients receiving anthracyclines [20].

The SGLT2 inhibitors not only protect the heart from the harmful effects of DOX but also do so without interfering with the anti-cancer mechanism of anthracyclines. DOX acts by generating reactive oxygen species (ROS), interfering with DNA synthesis, inhibiting topoisomerase II, and inducing apoptosis, essential for its cytotoxic effect on cancer cells. SGLT2 inhibitors act on myocardial cells, reducing oxidative stress and inflammation responsible for heart damage. These effects make SGLT2 inhibitors valuable in cardio-oncology [21,22].

Recent research suggests that SGLT2 inhibitors may even have anti-cancer effects. For instance, one comprehensive review has shown that dapagliflozin and canagliflozin can reduce tumor growth in cancer models by inhibiting glucose metabolism and limiting tumor cells’ energy resources [23].

This review explores the complexity of interactions between anthracycline treatments, particularly DOX, and the challenges associated with their cardiotoxicity. It will discuss the mechanism of action of DOX and the significant cardiac risks that may arise from treatment. The concept of cardio-oncology will be emphasized, highlighting the importance of an integrated approach in managing patients who require both anti-cancer treatments and cardiac protection. Given the preclinical and clinical evidence supporting their efficacy in reducing DOX-induced cardiotoxicity, SGLT2 inhibitors will be analyzed as agents with cardioprotective effects, bringing this class of therapeutics to the forefront as a core element of cardio-oncology. This integrated approach could transform the way we manage cancer patients at increased risk of cardiotoxicity by providing an innovative and effective therapeutic strategy.

## 2. Methodology and Study Selection

To ensure a comprehensive and up-to-date synthesis, an extensive literature search was performed across major medical databases, including PubMed, the Cochrane Library, and Web of Science. The search strategy incorporated keywords relevant to the manuscript’s focus, such as “doxorubicin”, “anthracycline”, “cardiotoxicity”, “heart failure”, “risk assessment”, “cardioprotection”, “SGLT2 inhibitors”, and “cardio-oncology”. The search included clinical trials, observational studies, preclinical research, systematic reviews, and clinical guidelines addressing the mechanisms, risk stratification, surveillance, and current treatment of AIC, as well as the emerging role of SGLT2 inhibitors in cardio-oncology. Priority was given to high-quality, peer-reviewed publications and guidelines. Additional sources were identified through a manual review of reference lists from key articles.

## 3. Doxorubicin-Induced Cardiovascular Toxicity: Overview of Mechanisms and Challenges

**Figure 1:**
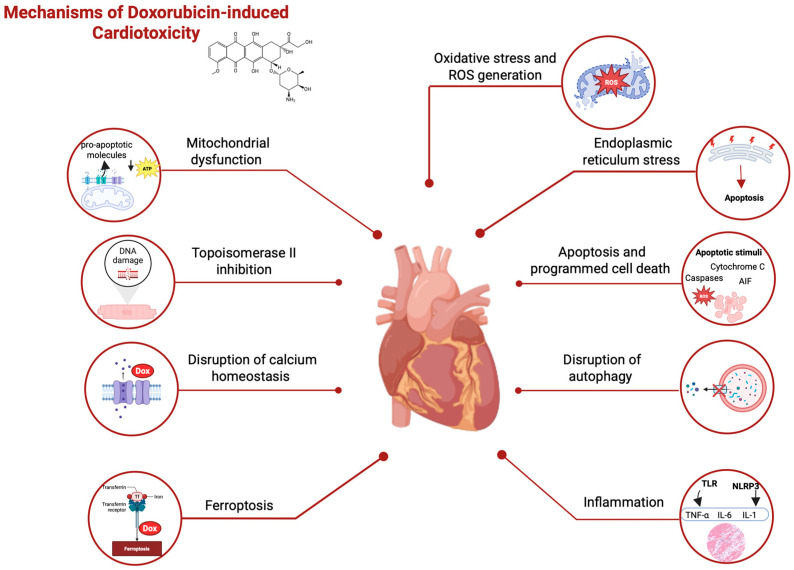
Mechanisms of Doxorubicin-induced Cardiotoxicity. AIF—apoptosis-inducing factor; ATP—adenosine triphosphate; Dox—doxorubicin; IL-1—interleukin 1; IL-6—interleukin 6; ROS—reactive oxygen species; TNF-α—tumor necrosis factor-alpha. Created in BioRender (https://BioRender.com/k6wpwmk).

The cardiovascular toxicity of DOX involves multiple complex and interconnected mechanisms. The main known mechanisms include oxidative stress and reactive oxygen species (ROS) generation [24], mitochondrial dysfunction [25], topoisomerase II inhibition [26], disruption of calcium homeostasis [27], endoplasmic reticulum stress [28], apoptosis and programmed cell death [29], ferroptosis [30], inflammation [31,32], and disruption of autophagy [33], as seen in Figure 1. Despite extensive research, the exact mechanisms and their interactions are not fully understood and require further study to develop more effective therapeutic strategies to prevent and treat DOX-induced cardiotoxicity.

Oxidative stress and the generation of ROS are considered the central mechanisms in DOX-induced cardiotoxicity. This complex process involves multiple pathways and molecular interactions and is linked to the dysregulation of antioxidants, following the destruction of subcellular structure and cell death [34]. DOX-induced ROS generation can be explained by the fact that DOX undergoes a one-electron reduction to the semiquinone form, catalyzed by several cellular enzymes, including nicotinamide adenine dinucleotide phosphate (NADPH) oxidases (NOXs) and uncoupled nitric oxide synthases (NOSs). Semiquinone can rapidly generate superoxide anions (•O_2_−) in the presence of oxygen. These superoxide anions can be converted to hydrogen peroxide (H_2_O_2_) by superoxide dismutase (SOD) [35]. Although relatively stable, H_2_O_2_ can generate highly reactive hydroxyl radicals (OH∙) via the iron-catalyzed Haber–Weiss reaction. These radicals can damage lipids, proteins, and DNA, contributing to cardiomyocyte injury [36]. DOX also increases the expression and activity of NOX2 and NOX4, amplifying ROS production and subsequent oxidative stress in cardiac myocytes [37]. DOX reduces the expression and activity of key antioxidant enzymes, including SOD, catalase (CAT), and glutathione peroxidase (GPx), compromising the ability of heart cells to manage increased oxidative stress [38]. In addition to ROS, DOX also induces the production of reactive nitrogen species (RNS). It stimulates the expression of inducible NOS (iNOS), leading to excessive nitric oxide (NO) production. NO can react with •O_2_− to form peroxynitrite (ONOO-), a powerful oxidant [34]. These effects cause mitochondrial dysfunction, disruption of calcium homeostasis, endoplasmic reticulum stress, and activation of cell death pathways, including ferroptosis. Collectively, these processes lead to cardiac myocyte loss, fibrosis, and, ultimately, cardiac dysfunction [39].

Mitochondria play a crucial role in cardiac cells, occupying up to a third of their total volume and providing the energy required for continuous myocardial contraction [40]. Disruption of mitochondrial permeability is a key mechanism in anthracycline-induced cardiotoxicity, particularly DOX, involving the opening of mitochondrial permeability transition pores (mPTPs). MPTP is a nonspecific channel located in the inner mitochondrial membrane with a complex structure and a crucial role in cell death. Its composition includes the Adenine Nucleotide Translocator (ANT) family localized in the mitochondrial inner membrane, F1Fo-ATPase (although its exact role in the structure of the mPTP is still debated), Bcl-2 family proteins with an important role in the regulation of apoptosis, and cyclophilin D (CypD), a mitochondrial matrix protein considered a prominent mediator of the mPTP [41,42]. Opening the mPTP releases pro-apoptotic molecules, such as cytochrome C, caspases, and apoptosis-inducing factor (AIF), associated with loss of mitochondrial membrane potential, disruption of calcium homeostasis, and decreased ATP production. Anthracycline-induced oxidative stress exacerbates mPTP opening, with reactive oxygen species (ROS) directly oxidizing mPTP components. In addition, anthracyclines disrupt calcium homeostasis, favoring calcium influx into the myocyte and Ca2+ release from the sarcoplasmic reticulum, potentiating mPTP opening [29,43]. These perturbations in mitochondrial permeability led to the activation of apoptotic pathways and ultimately to cardiac cell death, with progressive loss of cardiomyocytes contributing significantly to the development of cardiomyopathy and heart failure associated with anthracycline treatment [44].

Inhibition of topoisomerase II proved to be a central concern in the mechanism of DOX-induced cardiotoxicity. Topoisomerase II is an enzyme essential for DNA replication and transcription, and its inhibition by anthracyclines leads to the formation of stable complexes between the enzyme and DNA, which cause double helix breaks [45]. In cardiomyocytes, the predominant isoform is topoisomerase IIβ, inhibition of which leads to activation of cell death pathways and mitochondrial dysfunction. This process generates reactive oxygen species (ROS), disrupting cellular energy metabolism [46]. Inhibition of topoisomerase II also affects the expression of genes involved in mitochondrial biogenesis and energy metabolism, contributing to long-term cardiac dysfunction. Understanding this mechanism has led to the development of cardioprotective strategies, such as using liposomal formulations of anthracyclines [47] or novel derivatives of DOX (e.g., GPX-150, camsirubicin) that selectively target topoisomerase IIβ [48] to eliminate cardiotoxic side-effects.

Anthracyclines stimulate the release of proinflammatory cytokines, including tumor necrosis factor-alpha (TNF-α), interleukin-1 beta (IL-1β), and interleukin-6 (IL-6), by activating the NLRP3 inflammasome and TLR2/TLR4 signaling pathways [32]. The resulting chronic inflammation contributes to oxidative stress, apoptosis, and fibrosis, processes that lead to cardiomyocyte loss and maladaptive ventricular remodeling. Studies have shown that these inflammatory processes are closely linked to the progression of anthracycline-induced heart failure, and the severity of inflammation may be influenced by factors such as cumulative dose, patient age, and associated comorbidities [10]. A detailed understanding of the inflammatory mechanisms involved in anthracycline cardiotoxicity opens new therapeutic perspectives, including cytokine receptor blockers [49] or NLRP3 inflammasome inhibitors [50], to prevent progressive myocardial damage and improve the prognosis of cancer patients.

Understanding these complex mechanisms in anthracycline-induced cardiotoxicity is crucial for developing cardioprotective strategies and improving outcomes in cancer patients.

## 4. Risk Assessment and Surveillance of Anthracycline Cardiotoxicity

Predicting anthracycline-induced cardiotoxicity (AIC) in cancer patients is a complex and crucial challenge in modern medical practice. Early identification of high-risk patients allows the implementation of tailored prevention and monitoring strategies to minimize adverse effects on cardiac function without compromising the efficacy of cancer treatment [51]. Several major risk factors may increase a patient’s susceptibility to cardiotoxicity during treatment with anthracyclines, particularly DOX. These factors can be divided into categories such as cardiovascular history (pre-existing cardiovascular disease or cardiovascular risk factors), treatment-related factors (cumulative anthracycline dose, concomitant administration of other cardiotoxic therapies, or prior mediastinal radiotherapy), and patient characteristics (extreme age, female sex, or genetic factors) [52].

In terms of cardiovascular history, Hershman et al. showed that cancer patients with a record of heart disease, particularly those with multiple documented episodes of myocardial ischemia, atherosclerosis, or other heart conditions, are at significantly higher risk of developing congestive heart failure (CHF), following treatment with DOX [53]. Also, diabetes mellitus is a significant risk factor for cardiotoxicity induced by anthracyclines. Patients with diabetes have an increased predisposition to the development of diabetic cardiomyopathy, which makes them more vulnerable to the cardiotoxic effects of DOX [53]. Importantly, type 2 diabetes mellitus appears to be a significant predictor for DOX-induced late-onset HF but not for early-onset HF. This temporal distinction suggests that the mechanisms by which diabetes potentiates DOX cardiotoxicity may involve chronic and cumulative processes [54]. Dyslipidemia, although not assessed as an independent risk factor in AIC, has been included in studies demonstrating that patients with concomitant dyslipidemia and other cardiovascular risk factors (hypertension and diabetes) were significantly more likely to develop anthracycline-induced cardiomyopathy compared to cohorts without these comorbidities [55]. These observations suggest that an altered lipid profile, particularly in the context of a pre-existing metabolic syndrome, may amplify myocardial vulnerability to the cardiotoxic effects of anthracyclines, emphasizing the need for a holistic approach to cardiovascular risk assessment in oncologic patients who are candidates for anthracycline therapy [10].

The cumulative dose administered is a pivotal factor in the onset of AIC. This is a significant area of research, as evidenced by the substantial retrospective studies that have analyzed the dose–response relationship in the emergence of congestive heart failure (CHF) in patients treated with DOX. Swain et al.’s analysis of three clinical trials involving 630 doxorubicin-treated patients revealed compelling findings. Notably, a cumulative dose of 550 mg/m^2^ led to CHF in approximately 26% of patients, with over half of these cases experiencing a severe reduction in LVEF below 30% [56]. A study published by Von Hoff et al., which included 4018 DOX-treated patients, further supports the dose-dependent relationship. The incidence of cardiotoxicity was found to be 0.14% at a cumulative dose of 400 mg/m^2^, a figure that surged to 7% at the 550 mg/m^2^ dose [57]. These findings underscore the need for vigilant monitoring of the cumulative anthracycline dose. It is crucial to implement cardioprotective strategies when these dose thresholds are surpassed, as the risk of DOX-associated cardiomyopathy becomes significant at cumulative doses exceeding 400 mg/m^2^ and escalates notably at doses above 500 mg/m^2^.

Concomitant administration of other cardiotoxic therapies or prior mediastinal radiotherapy are significant risk factors for the development of DOX-induced cardiotoxicity. The combination of anthracyclines and trastuzumab (monoclonal antibody) in the treatment of breast cancer raises significant concerns about cardiotoxicity. A retrospective review of two large clinical trials (NSABP B-31 and NCCTG N9831) compared the incidence of symptomatic heart failure in patients treated with DOX and cyclophosphamide, with or without the addition of adjuvant trastuzumab. The results showed a more than four-fold increase in the risk of HF when trastuzumab was added to the standard chemotherapy regimen (2.0% vs. 0.45%) [58]. It should also be mentioned that the synergistic effect of mediastinal radiotherapy and DOX administration in oncological patients significantly amplifies the risk of cardiotoxicity. Patients receiving both treatments had an increased incidence of cardiac hospitalizations over 10 years, more than 20% higher than those receiving DOX alone [59].

Assessing gender differences in the prevalence of HF induced by DOX treatment has proven to be a significant challenge in medical research. Many studies have not performed rigorous comparative statistical analyses between male and female subgroups, leading to sampling bias [60]. The predominant use of male rodents in experimental studies investigating DOX-induced cardiotoxicity reflects a broader issue in biomedical research, potentially limiting our understanding of sex differences regarding cardiotoxicity prediction in cancer patients [61]. A preclinical study published in 2015 by Moulin et al. showed important sex differences in DOX-induced cardiotoxicity. After 7 weeks of treatment, the males showed significant signs of heart failure, including ascites and pleural effusion. Echocardiography showed a significant reduction in LVEF in treated males, from 87.5% to 55.9% (*p* < 0.0002), whereas in females, LVEF was only moderately affected, from 86.3% to 79.0% (*p* < 0.01). Also, significant changes in left ventricular systolic internal diameter and cardiac output were only observed in treated males. These results, in conjunction with mortality data, indicate a significant sex difference in the development of DOX-induced cardiomyopathy, with more severe impairment of cardiac function in treated male rats [62].

Research on gender differences regarding DOX-induced cardiotoxicity in humans is limited, with few studies including adult participants and contrasts between results observed in adult and pediatric oncology patients. Studies indicate an increased risk among young girls compared to boys of the same age [63,64]. This higher susceptibility could theoretically be explained by the low estrogen levels in the female body before puberty [65]. The lack of cardioprotective effects of estrogen during this period could expose the myocardium to increased vulnerability to the toxic effects of doxorubicin.

Genetic factors, particularly gene polymorphisms involved in DOX metabolism or antioxidant defense mechanisms, may influence individual susceptibility to cardiotoxicity. Genome-wide and candidate gene-based association studies revealed 80 genes with single-nucleotide polymorphisms (SNPs) significantly associated with AIC [66]. A recent study published by Fonoudi et al. in 2024 validates 38 genes related to AIC using CRISPR/Cas9 knockout in human induced pluripotent stem cell-derived cardiomyocytes (iPSC-CMs), providing insights for future studies on DOX cardiotoxicity variant associations and potential targets for cardioprotective drug development [67].

### 4.1. Risk Stratification Before Anthracycline Treatment

Cardiotoxicity risk stratification in oncologic patients before initiation of anthracycline therapy, particularly DOX, is a crucial step in cardio-oncology [68]. According to the ESC 2022 Cardio-Oncology Guidelines, a cardiovascular risk assessment should be performed on all patients diagnosed with cancer who are to receive treatments with significant cardiotoxic potential [10]. The ESC 2022 Guidelines recommend using the HFA-ICOS Cardio-Oncology cardiovascular risk assessment tool, which combines the following parameters: previous history of cardiovascular disease, cardiac biomarkers, age, cardiovascular risk factors, previous cardiotoxicity treatment, and lifestyle risk factors. It also considers the type and planned cumulative dose of anthracyclines and the concomitant administration of other potentially cardiotoxic agents [69].

HFA-ICOS results rank cancer patients into three categories: low, moderate, and high or very high risk. This stratification guides subsequent patient management decisions. For those at low risk, routine oncologic monitoring is recommended. Moderate-risk patients may require further cardiologic evaluation. A multidisciplinary approach is needed for patients at high or very high risk, including cardiology consultation, careful consideration of the risk-benefit ratio of potentially cardiotoxic oncologic therapy, and implementation of personalized cardioprotective strategies. This differentiated approach optimizes patient care, balancing the need for effective oncology treatment while preventing cardiovascular complications [10,70,71].

### 4.2. Cancer Therapy–Related Cardiac Dysfunction Definition

The 2022 ESC Guidelines on cardio-oncology define cancer therapy-related cardiac dysfunction (CTRCD) along a spectrum of severity, classified as either symptomatic or asymptomatic. Symptomatic CTRCD, representing heart failure (HF), ranges from mild symptoms to very severe HF requiring inotropic support, mechanical circulatory support, or consideration of transplantation. Asymptomatic CTRCD is defined by decreased left ventricular ejection fraction (LVEF) and global longitudinal strain (GLS) from baseline, with more significant reductions indicating greater severity. Specifically, a new LVEF reduction to less than 40% signifies severe asymptomatic CTRCD. A reduction of 10 percentage points to an LVEF between 40 and 49%, or a smaller decline accompanied by a relative GLS decline exceeding 15% or a new rise in cardiac biomarkers, is considered moderate. Mild asymptomatic CTRCD is characterized by preserved LVEF (≥50%) with a relative GLS decline exceeding 15% and/or a new increase in cardiac biomarkers [10].

### 4.3. Ongoing Surveillance During Anthracycline Treatment

**Figure 2:**
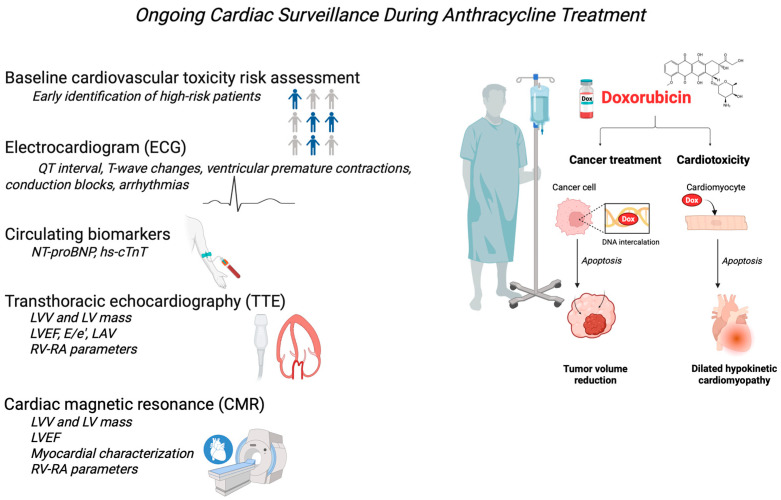
Summary of methods for monitoring anthracycline-induced cardiotoxicity (AIC). CMR—cardiac magnetic resonance; ECG—electrocardiogram; hs-cTnT—high-sensitivity cardiac troponin T; LAV—left atrium volume; LVEF—left ventricular ejection fraction; LVV—left ventricular volume; NT-proBNP—N-terminal pro-B-type natriuretic peptide; RV-RA—right ventricle-right atrium. Created in BioRender (https://BioRender.com/d9h9ht3).

Continuous cardiovascular monitoring of patients during anthracycline therapy is essential to cardio-oncology management (Figure 2). It aims to detect possible therapy-induced cardiac changes early and prompt intervention to prevent severe complications [10].

The electrocardiogram (ECG) plays an important role in this surveillance, being a non-invasive, accessible, and reproducible investigation. The importance of the ECG in anthracycline treatment derives from its ability to reveal subtle changes in QT interval, T-wave changes, ventricular premature contractions, or conduction blocks, which may predict cardiotoxicity [72,73]. Although the ECG is not a direct indicator of myocardial dysfunction, its changes may suggest the need for further investigations, such as echocardiography or determination of cardiac biomarkers. Current cardio-oncology guidelines, including the ESC 2022 Guidelines, have a class I indication that an ECG be performed before initiation of anthracycline therapy to serve as a baseline and allow comparison with subsequent ECGs [10].

Circulating biomarkers are a sensitive and cost-effective strategy for monitoring patients on anthracycline therapy. Troponins and natriuretic peptides (NPs) have been intensively studied in this context [74,75]. Highly sensitive troponin assays allow early detection of minimal myocardial injury. However, the interpretation of results must consider the variability of assay methods, gender-specific thresholds, and the time interval since the anthracycline administration [76,77]. A study published by Shafi et al. concluded that early release of troponin I (TnI) after anthracycline chemotherapy in breast cancer patients is a significant predictor of left ventricular systolic dysfunction (LVSD), such that cardiotoxicity occurred in 50% of patients with elevated TnI levels and only in 1.5% of patients with normal TnI [78]. Another prospective study, enrolling 68 patients undergoing DOX treatment (220–500 mg/m^2^), found that 22.1% developed anthracycline-induced cardiomyopathy after 6 months. Elevated plasma levels of high-sensitivity cardiac troponin T (hs-cTnT) and N-terminal pro-B-type natriuretic peptide (NT-proBNP) at 3 and 6 months, along with their percentage increases, were predictive of AIC development. Specifically, NT-proBNP levels greater than 118.5 pg/mL and hs-cTnT levels above 0.008 pg/mL, combined with an increase greater than 20% at 3 months, were independent predictors of AIC at 6 months, highlighting their potential utility in early risk stratification [79]. Recent research showed that elevated levels of hs-cTnT ≥ 0.019 ng/mL and NT-proBNP ≥ 31.1 pg/mL measured at 3 months post-anthracycline treatment, alongside relative reductions in left ventricular global longitudinal strain (LV GLS ≥ 6.5%) and left atrial reservoir strain (LASr ≥ 7.5%) at the same time point, have emerged as independent predictors of cardiotoxicity development. These findings, derived from a prospective observational cohort study, highlight the utility of incorporating these biomarkers and echocardiographic parameters into a risk score, along with clinical variables, to identify patients at increased risk of developing anthracycline-induced cardiotoxicity within a 2-year follow-up period [80]. Converging data from multiple studies indicate that serial determination of cardiac troponin and NT-proBNP should be integrated into the algorithm for early diagnosis of AIC. These markers, reflecting myocyte injury and hemodynamic stress associated with heart failure, provide valuable information to identify patients at increased risk of subclinical cardiac dysfunction, thus allowing therapeutic interventions [81,82,83,84,85].

Recent research has investigated several new biomarkers beyond troponin and NT-proBNP to refine the predictability of anthracycline-induced cardiotoxicity. These markers target specific pathophysiological processes involved in cardiac damage, such as inflammation, oxidative stress, and fibrosis. They include galectin-3 [86], a mediator of myocardial fibrosis; myeloperoxidase (MPO) [87], an indicator of oxidative stress and neutrophil activation; growth differentiation factor-15 (GDF-15) [88], associated with cellular stress and mitochondrial dysfunction; and microRNA (miRNA) molecules [89,90,91], which regulate gene expression and may reflect adaptive or maladaptive responses of the myocardium to treatment. Although preliminary studies suggest the potential of these new markers for early identification of patients susceptible to cardiotoxicity, further validation in large cohorts of patients and the establishment of standardized threshold values are essential before their implementation in routine clinical practice [92].

Transthoracic echocardiography (TTE) plays a central role in detecting and monitoring AIC, providing a non-invasive assessment of cardiac structure and function [93]. The key parameter assessed by TTE in the context of cardiotoxicity is the LVEF, long considered the ‘gold standard’ for evaluating systolic function [94]. However, TTE also allows the assessment of GLS, a more sensitive measure of myocardial function than LVEF, and can detect subclinical changes in ventricular contractility. GLS is particularly useful in the early identification of cardiotoxicity before the LVEF falls significantly [95]. In addition, ETT provides information about diastolic function, which anthracyclines may affect. Diastolic function parameters such as E/e’ ratio, lateral and septal e’ velocities, and left atrial volume (LAV) [96] contribute to the global assessment of cardiac function.

Cardiac magnetic resonance (CMR), while not routinely employed in standard cancer patient monitoring due to accessibility constraints, cost considerations, and patient compliance issues, offers superior accuracy in assessing left ventricular ejection fraction (LVEF) and biventricular parameters, along with the unique ability to characterize myocardial tissue, leading to its increasing adoption in clinical research [10,97,98]. CMR can identify microvascular obstruction, iron overload, and diffuse interstitial fibrosis through advanced techniques like T1 and T2 mapping and quantify focal fibrosis using late gadolinium enhancement [99,100]. In clinical practice, CMR is primarily reserved for cases where echocardiography provides suboptimal data or when myocarditis is suspected. It is also valuable for diagnosing pericardial disease, which is common in patients receiving mediastinal radiation or anthracyclines [98,99]. The selection of cardiac imaging modality should be based on local expertise and availability, with consistent use of the same technique throughout treatment to minimize variability [101].

## 5. Doxorubicin-Induced Cardiovascular Toxicity: Current and Future Treatments

Despite progress in understanding the mechanisms underlying DOX cardiotoxicity, developing effective strategies for its prevention and treatment remains a priority in cardio-oncology. In recent years, significant efforts have been made to identify and validate cardioprotective therapies that allow anthracyclines to be used under improved safety conditions [10].

Dexrazoxane was approved for clinical use after clinical trials demonstrated its ability to prevent cardiotoxicity without compromising the effectiveness of chemotherapy [102,103]. Dexrazoxane acts through several mechanisms, including inhibition of free iron and reduction in oxidative stress, which help protect the myocardium from the toxic effects of anthracyclines [104]. Dexrazoxane inhibits the formation of the doxorubicin-topoisomerase IIβ complex, thereby reducing DOX-induced apoptosis and cell necrosis [105]. Clinical studies have shown that dexrazoxane significantly reduces the risk of HF and cardiac events in anthracycline-treated patients. A study published by Chow et al. enrolled 195 participants, with a mean age of 28.8 ± 5.3 years at enrollment and a mean of 18.1 ± 2.7 years since cancer diagnosis. Participants received average DOX doses of approximately 300 mg/m^2^. The results showed that the dexrazoxane-treated group had improved left ventricular (LV) systolic function and significantly reduced BNP and NT-proBNP levels. These benefits were more pronounced in women and those receiving higher doses of DOX (≥250 mg/m^2^). Multivariable analyses confirmed that dexrazoxane is associated with improved LV function and lower levels of myocardial stress biomarkers, becoming a patient-centered decision in preventing anthracycline-induced cardiotoxicity [106]. Dexrazoxane is administered as an intravenous infusion before each dose of DOX at a 10:1 ratio. Dexrazoxane infusion is usually given within 15–30 min, followed by doxorubicin at least 30 min later [107]. Initially, the use of dexrazoxane was limited by concerns about its potential impact on the efficacy of oncologic treatment and the risk of secondary malignancies [108]. Subsequent studies have not confirmed these risks, as observed in research published by Asselin et al. [109].

Statins, known for their hypolipidemic effects, have been investigated for cardioprotective potential in preventing AIC [110]. Preclinical studies, such as the one published by Oh et al. [111] in 2020, show that atorvastatin exerts a cardioprotective effect against DOX-induced cardiotoxicity via a transcriptional network involving FOXO1, STAT3, and Sp1. DOX was found to suppress survivin expression, a cell survival factor, by activating FOXO1, blocking the STAT3/Sp1 complex, which is required for survivin transcription. Atorvastatin inhibited FOXO1, stabilizing the STAT3/Sp1 complex and restoring survivin expression, leading to improved cardiac function in an animal model. These results suggest a novel pathophysiologic mechanism by which atorvastatin could prevent DOX-induced cardiotoxicity, highlighting its therapeutic potential in cardio-oncology [111]. Another preclinical study investigated the effects of fluvastatin (FV) in preventing doxorubicin-induced cardiac and renal toxicity. The results showed that FV exerts a protective effect by reducing lipid peroxidation and upregulating the expression of genes involved in the inflammatory and apoptotic response, such as iNOS, eNOS, nuclear factor kappa-B (NF-κB), and Caspase-3. These changes attenuated DOX-induced toxic mechanisms, suggesting that FV might be a helpful agent in preventing renal and cardiac toxicity in patients receiving DOX chemotherapy [112]. The PREVENT and STOP-CA trials investigated the role of atorvastatin in preventing anthracycline-induced cardiotoxicity but reported different results. The PREVENT trial, involving 279 patients with breast cancer or lymphoma treated with anthracyclines, showed no significant difference in LVEF between the atorvastatin 40 mg and placebo-treated groups at 24 months [113]. In contrast, the STOP-CA trial, involving 300 anthracycline-treated lymphoma patients, showed a decreased incidence of LV systolic dysfunction in patients receiving atorvastatin 40 mg compared to those receiving placebo. Only 9% of patients in the atorvastatin group experienced a decrease in LVEF by ≥10% to below 55%, compared with 22% in the placebo group over the 12-month study period [114]. These differences in outcomes may be attributable to variability in the doses of anthracyclines used, study population, and duration of follow-up, with STOP-CA including higher doses of anthracyclines and showing more precise cardioprotective benefits.

Renin–angiotensin system (RAS) inhibitors, including angiotensin-converting enzyme inhibitors (ACEIs) and angiotensin receptor blockers (ARBs), have been investigated for their cardioprotective potential in preventing anthracycline-induced cardiotoxicity. Various clinical trials have evaluated the effect of angiotensin antagonists, such as candesartan, enalapril, and ramipril, in preventing anthracycline-induced cardiotoxicity [115]. For example, the PRADA trial investigated the effect of candesartan and metoprolol (β-blocker) in preventing AIC in breast cancer patients. The results showed that candesartan attenuated a slight decline in GLS. Also, metoprolol reduced the increase in cardiac troponin I during anthracycline treatment, suggesting a protective effect on acute myocardial injury [116]. The SAFE trial, which included 174 women, reported that ramipril or combining ramipril with bisoprolol protected against anthracycline-induced LVEF decline [117]. However, experts believe that more extensive randomized trials are needed to confirm these results and assess the impact on relevant clinical endpoints (e.g., mortality, incidence of HF). It is also important to better characterize high-risk populations and conduct studies in these groups to optimize cardioprotective strategies [115].

A multicenter randomized, placebo-controlled, multicenter trial called PRADA II (Prevention of Cardiac Dysfunction During Therapy for Breast Cancer) is ongoing to evaluate the preventive cardioprotective effect of sacubitril-valsartan during (neo)adjuvant therapy in breast cancer patients who are to receive anthracycline-containing therapy [118].

The ESC 2022 guidelines for cardio-oncology recommend strategies to prevent cardiovascular toxicity associated with oncologic therapies, including the use of dexrazoxane, liposomal anthracyclines, ACEIs/ARBs, beta-blockers and statins for adult cancer patients receiving anthracycline therapy who are at high or very high risk of cardiovascular toxicity (Class IIa recommendation) [10].

The prevention of AIC is an active and dynamic field with numerous ongoing clinical trials exploring various cardioprotective strategies and novel therapeutic agents. Recently, research has emerged exploring the potential of SGLT2 inhibitors as protective agents against AIC.

## 6. SGLT2 Inhibitors: A Journey from Discovery to Cardio-Oncology

Sodium-glucose co-transporter 2 (SGLT2) inhibitors, also known as gliflozins, were discovered following the isolation of phlorizin from the bark of apple trees in 1835 and were initially studied for treating fever [119]. Later, in 1962, research demonstrated that phlorizin competitively inhibits glucose and sodium transport in the renal proximal tubule [120]. The development of synthetic SGLT2 inhibitors began in the 1990s when Japanese pharmaceutical companies created molecules such as T-1095, which increases urinary glucose excretion in diabetic animal models, thereby decreasing blood glucose concentration [121]. This class of drugs has been intensively studied, and later, selective molecules developed, such as canagliflozin, dapagliflozin, and empagliflozin, which predominantly block SGLT2, minimizing the adverse effects associated with SGLT1 inhibition [122].

The original purpose of SGLT2 inhibitors was to manage type 2 diabetes mellitus (T2DM) by lowering blood glucose through urinary glucose excretion. Later clinical trials have demonstrated significant benefits for cardiovascular and kidney health, in addition to glycemic control. Trials such as CREDENCE (Impact of Canagliflozin on Renal and Cardiovascular Outcomes in Patients with Diabetic Nephropathy) [123], VERTIS CV (Cardiovascular Outcomes with Empagliflozin in Patients with T2DM) [124], EMPA-REG OUTCOME (Empagliflozin Cardiovascular Outcome events trial in T2DM) [125], and DECLARE-TIMI 58 (Dapagliflozin and Cardiovascular Outcomes in Patients with T2DM) [126] have shown that SGLT2 inhibitors reduce the risk of major cardiovascular events, including cardiovascular death and heart failure (HF), and improve renal outcomes in patients with diabetes and chronic kidney disease (CKD).

According to the ESC 2023 Focus Update of the 2021 ESC Heart Failure Guidelines and the AHA/ACC/HFSA 2022 Guidelines, SGLT2 inhibitors are recommended as Class I for all forms of heart failure: HF with reduced ejection fraction (HFrEF), HF with mildly reduced EF (HFmrEF), and HF with preserved EF (HFpEF) [15,127]. They are recognized as essential components of HF-directed therapy due to their effectiveness in reducing hospitalizations and cardiovascular mortality. Moreover, they are indicated for slowing the progression of chronic kidney disease (CKD) and preventing major cardiovascular events in patients with diabetes mellitus or albuminuria (Class I recommendation for treating adults with CKD using an SGLT2 inhibitor for an estimated glomerular filtration rate of ≥20 mL/min per 1.73 m^2^ and a urine albumin-to-creatinine ratio of ≥200 mg/g) [128].

Various studies investigate the potential role of SGLT2 inhibitors in preventing cardiotoxicity caused by anthracyclines. The cardioprotective properties of SGLT2 inhibitors in patients undergoing anthracycline treatment are complex, presenting new opportunities for their use in cardio-oncology. Firstly, SGLT2 inhibitors influence inflammatory responses and oxidative stress associated with chemotherapy-related cardiac injury by decreasing myocardial inflammatory mediators, including TNF-α, NF-κB, and interleukins (ILs) [129]. In a recent study published by Quagliariello et al., cardiomyocytes exposed to dapagliflozin and DOX were shown to have significantly lower levels of cytokines and NLRP3-Myd88 compared to those exposed to DOX alone. Dapagliflozin also reduced the p65/NF-κB expression induced by DOX and preserved the cardiac and renal tissue microstructure. The study also showed that dapagliflozin has systemic anti-inflammatory effects, significantly reducing inflammatory markers such as high-sensitivity C-reactive protein (hs-CRP), IL-1, and Galectin-3 [130]. A crucial mechanism through which SGLT2 inhibitors provide cardioprotection is reducing the production of reactive oxygen species (ROS), which decreases cardiomyocyte apoptosis via activation of the phosphatidylinositol 3-kinase (PI3K)/protein kinase B (AKT) signaling pathway essential for cardiomyocyte survival [131]. Dapagliflozin may also modulate STAT3 signaling, which plays a role in various cardioprotective processes, as a promoter of the metabolic network [132]. Additionally, SGLT2 inhibitors promote autophagy, the primary cellular recycling mechanism. In experimental models, empagliflozin enhances autophagic flux and decreases the buildup of autolysosomes, thereby protecting the heart from cardiotoxic effects [133]. Ferroptosis, induced by anthracyclines, is a non-apoptotic, iron-dependent cell death type. SGLT2 inhibitors have demonstrated the ability to attenuate cardiomyocyte injury by inhibiting ferroptosis through the MAPK signaling pathway [134,135]. Moreover, SGLT2 inhibitors influence metabolic pathways, such as ketone body production, which is vital for cardiac energy management [136]. However, the EMPA-VISION trial stated that empagliflozin did not enhance cardiac energetics or alter circulating ketone body levels in patients with HF, regardless of EF status, compared to placebo [137]. Although there is no conclusive evidence that SGLT2 inhibitors’ effects on HF involve ketone metabolism, the presence of ketones following SGLT2 inhibition may still offer therapeutic benefits for cardiovascular protection, probably due to ketones’ ability to activate nutrient deprivation signals rather than serving as a primary energy source for ATP synthesis [138].

**Figure 3:**
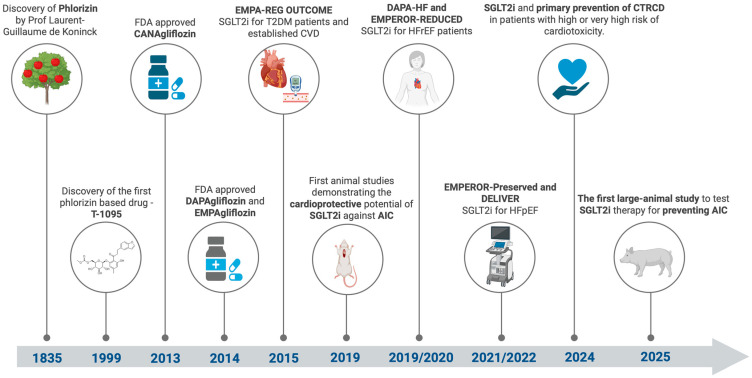
The journey of SGLT2 inhibitors—from their initial discovery and approval for diabetes management to their emerging dual role in cardio-oncology. AIC = anthracycline-induced cardiotoxicity; CTRCD = cancer therapy-related cardiac dysfunction; CVD = cardiovascular disease; HFrEF = heart failure with reduced ejection fraction; HFpEF = heart failure with preserved ejection fraction; SGLT2i = sodium-glucose co-transporter 2 inhibitors; T2DM = type 2 diabetes mellitus. Created in BioRender (https://BioRender.com/qjt1u0d).

Using SGLT2 inhibitors during anthracycline therapy has shown significant advantages, including preserving cardiomyocyte structure, reducing myocardial fibrosis, and enhancing cardiac contractility. A comprehensive review published in 2024 captures the cardioprotective effects of SGLT2 inhibitors, which ultimately lead to improved cardiac remodeling, as evidenced by reduced left ventricular mass and improved LVEF [139]. Figure 3 provides a comprehensive timeline of key milestones, showcasing the pivotal discoveries, clinical advancements, and expanding therapeutic applications of SGLT2 inhibitors.

The latest studies have revealed that SGLT2 inhibitors could enhance cancer treatment outcomes by reducing tumor growth and improving prognosis through mechanisms independent of their glucose-lowering effects [140,141].

## 7. Preventing Anthracycline Cardiotoxicity Using SGLT2 Inhibitors in Preclinical Models

Preclinical studies in animal models have consistently demonstrated that SGLT2 inhibitors (e.g., empagliflozin, dapagliflozin) attenuate DOX-induced deterioration in cardiac function. In mice, empagliflozin administration reduced ventricular hypertrophy and interstitial fibrosis. It maintained left ventricular ejection fraction (LVEF) close to basal levels, even at high cumulative doses of DOX (single intraperitoneal injection of DOX-HCl at 15 mg/kg) [142]. Besides improvements in LVEF, fractional shortening [21,143], and inhibiting LV remodeling [144], empagliflozin showed protection against DOX-induced prolongation of QT and corrected QT intervals [145]. One study that used a large animal model to test SGLT2 inhibitor therapy to prevent AIC showed that empagliflozin (20 mg/day) prevented cardiomyocyte atrophy and reduced levels of DNA damage markers (TUNELs) in the heart of DOX-treated pigs (6 triweekly intravenous DOX injections of 1.8 mg/kg each) compared to the control group [146].

Animal model studies also demonstrate that dapagliflozin provides cardioprotective benefits alone [132,147,148] or combined with other drugs of choice in treating heart failure. In murine models, administration of dapagliflozin (1 mg/kg/day) in combination with sacubitril/valsartan (34 mg/kg/day) improved survival rates in an acute doxorubicin experiment (single dose of 15 mg/kg DOX). It also proved that low-dose sacubitril/valsartan, in combination with SGLT2 inhibitors, exhibits a favorable effect on hemodynamic parameters and improves myocardial metabolic function by activating the PPAR signaling pathway [149]. Dapagliflozin also significantly reduced the accumulation of reactive oxygen species (ROS) and the expression of proinflammatory cytokines in cardiomyocytes of DOX-treated mice, protecting against fibrosis and ventricular remodeling [150,151,152].

Following groundbreaking research that established the cardioprotective effects of SGLT2 inhibitors in HF, numerous studies using animal models have highlighted their potential to reduce cardiotoxicity caused by DOX, as seen in Table 1. Preclinical studies were identified systematically across major medical databases using related subject keywords. Studies were selected according to their investigation of SGLT2 inhibitors in animal models of DOX-induced cardiotoxicity. Priority was given to peer-reviewed publications demonstrating cardioprotective efficacy (e.g., improved echocardiographic parameters and ECG results; reduced cardiac biomarkers and myocardial fibrosis). Studies lacking control groups or insufficient methodological detail were excluded.

## 8. Preventing Anthracycline Cardiotoxicity Using SGLT2 Inhibitors in Clinical Models

Recent clinical trials emphasize the role of SGLT2 inhibitors in reducing anthracycline-induced cardiotoxicity (AIC), particularly DOX. A meta-analysis of four observational studies (*n* = 5590 patients) revealed that anthracycline-treated individuals receiving SGLT2 inhibitors experienced a significantly lower risk of all-cause mortality (RR = 0.55, 95% CI: 0.39–0.77), while heart failure incidence showed a non-significant downward trend (RR = 0.67, 95% CI: 0.40–1.41) [156]. These findings align with more extensive analyses, such as a 2025 study (*n* = 88,273), which reported a 71% reduction in new heart failure diagnoses among cancer patients receiving anthracycline-based chemotherapy and SGLT2 inhibitors [157].

Table 2 overviews key clinical studies evaluating SGLT2 inhibitors and their ability to reduce cardiotoxic effects associated with anthracycline-based cancer treatments. The inclusion criteria focused on human studies (randomized controlled trials, observational cohorts, or case series) reporting cardiovascular outcomes in patients receiving anthracyclines with SGLT2 inhibitors. Studies were prioritized if they provided data on cardiac function, biomarkers, or clinical endpoints. While these results highlight the potential of SGLT2 inhibitors in cardio-oncology, they need further validation through large-scale randomized trials to determine optimal dosing in the non-diabetic cancer population and establish causal relationships.

## 9. Conclusions

The expanding role of SGLT2 inhibitors in cardio-oncology marks the dawn of a new era, recasting them from glycemic control agents into cardiac protection tools during anthracycline therapy. Current preclinical and observational studies highlight their ability to reduce anthracycline-induced cardiotoxicity via pleiotropic mechanisms, such as shifting cardiac metabolism towards ketone body utilization, reducing inflammation, and decreasing oxidative stress. Moreover, recent meta-analyses indicate significant declines in overall mortality and risk of HF in patients using SGLT2 inhibitors. However, these results are limited by the observational nature of the available data and the predominant focus on diabetic populations.

SGLT2 inhibitors represent a promising opportunity in cardio-oncology. Their favorable safety profile and pleiotropic benefits position them as key agents in preventing cardiovascular morbidity associated with oncologic treatments.

## Figures and Tables

**Table 1:** Preclinical studies supporting the cardioprotective effects of SGLT2 inhibitors against AIC.

Author/Year	Species	DOX Dosage and Administration	SGLT2 Inhibitor	Cardiovascular Assessment
Oh Et Al. (2019) [19]	Male C57BL/6J (B6J)	15 mg/kg i.p. single dose	EMPA 300 mg/kg in NCD for 2 weeks	↑ Fractional shortening; ↓ LV hypertrophy; ↓ Perivascular and interstitial fibrosis;
Maurea et al. (2019) [153]	Male C57BL6 mice	2.25 mg/kg/day i.p.	EMPA 10 mg/kg/day	Prevented GLS reduction;
Sabatino et al. (2020) [142]	Male C57Bl/6 mice	15 mg/kg i.p. single dose/4 mg/kg/week for 5 weeks	EMPA 10 mg/kg/day	Improved BP values and LV systolic function; ↓ Myocardial fibrosis;
Wang et al. (2020) [133]	Male C57BL/6 mice	5 mg/kg/week for 4 weeks i.v.	EMPA 0.05 mg/kg/day for 5 weeks	↑ Fractional shortening; ↑ Cardiac contractility; ↓ Myocardial fibrosis; ↓ BNP and cTnT;
Quagliariello Et Al. (2021) [21]	Female C57Bl/6 mice	2.17 mg/kg/day for 7 days	EMPA 10 mg/kg/day for 10 days	Attenuated fractional shortening and LVEF decline;
Baris et al. (2021) [145]	Male Sprague Dawley rats	18 mg/kg for 7 days i.p. cumulative dose	EMPA 10 mg/kg for 14 days	↑ LVEF; ↓ QTc interval and myofibril loss;
Chang et al. (2022) [150]	Male Sprague Dawley rats	5 mg/kg/week for 4 weeks i.p.	DAPA 10 mg/kg/day for 6 weeks	↑ Fractional shortening; ↑ LVEF; ↓ Hemodynamic changes in echo parameters; ↓ Myocardial fibrosis;
Hsieh et al. (2022) [132]	Sprague Dawley rats	3 mg/kg/week for 4 weeks i.p.	DAPA 0.1 mg/kg/day for 4 weeks	Attenuated LVEF decline and cardiac hypertrophy;
Satyam et al. (2023) [154]	Adult Wister rats	20 mg/kg i.p. single dose	DAPA 0.9 mg/kg/day for 8 days	Attenuated CK-MB increase and pathological ECG changes (e.g., QTc prolongation);
Chen et al. (2023) [143]	Male Sprague Dawley rats	2.5 mg/kg/twice a week for 4 weeks i.p.	EMPA 30 mg/kg/day for 4 weeks	↑ LVEF and fractional shortening; ↓ NT-proBNP and cTnT;
Quagliariello Et Al. (2024) [130]	Female C57Bl/6 mice	2.17 mg/kg/day i.p.	DAPA 10 mg/kg/day	Attenuated LVEF decline and cardiac strain impairment;
Chang et al. (2024) [155]	C57Bl/6 mice	5 mg/kg/week for 4 weeks	EMPA 10 mg/kg/day for 5 weeks	Attenuated LVEF and fractional shortening decline;
Medina-Hernández et al. (2025) [146]	Female large white pigs	6 triweekly i.v. injections (1.8 mg/kg each)	EMPA 10/20 mg/day	Improved LVEF and cardiac energetics in a dose-dependent manner;
Maurea et al. (2025) [152]	Female C57Bl/6 mice	2.17 mg/kg/day i.p. for 10 days	DAPA 12 mg/kg for 10 days	↑ LVEF and fractional shortening;

BNP = B-type natriuretic peptide; BP = blood pressure; cTnT = cardiac troponin T; DAPA = dapagliflozin; EMPA = empagliflozin; GLS = global longitudinal strain; i.p. = intraperitoneal; i.v. = intravenous; LV = left ventricular; LVEF = left ventricular ejection fraction; NCD = normal chow diet; NT-proBNP = N-terminal pro-B-type natriuretic peptide; QTc = corrected QT; ↓ = decreases; ↑ = increases.

**Table 2:** Clinical studies supporting the cardioprotective effects of SGLT2 inhibitors against AIC.

Author/Year	Population	Final Cohort	SGLT2 Inhibitor	Key Findings
Gongora et al. (2022) [20]	Cancer patients diagnosed with DM prior to anthracycline treatment	32 SGLT2i recipients/96 SGLT2i non-recipients	EMPA 16 pts CANA 11 pts DAPA 5 pts	Patients on SGLT2 inhibitors: ↓ Cardiac events post anthracycline therapy; ↓ HF admissions; ↓ Rate of cardiac dysfunction; No new cases of AIC observed;
Chiang et al. (2023) [158]	Adult patients with T2DM diagnosed with cancer	878 SGLT2i recipients/7556 SGLT2i non-recipients	EMPA CANA DAPA	Patients on SGLT2 inhibitors: ↓ HF admissions; ↑ Overall survival;
Hwang et al. (2023) [159]	Adult patients newly diagnosed with cancer undergoing anthracycline-based chemotherapy	7800 non-DM/779 SGLT2i recipients/2337 SGLT2i non-recipients	EMPA CANA DAPA	Patients on SGLT2 inhibitors: ↓ Cardiovascular composite outcome (stroke, MI, HF admissions, death);
Abdel-Qadir et al. (2023) [160]	Patients over 65 years old with DM and no prior HF undergoing anthracycline treatment	99 SGLT2i recipients/834 SGLT2i non-recipients	EMPA CANA DAPA	Patients on SGLT2 inhibitors: ↓ HF hospitalizations;
Avula et al. (2024) [161]	Patients aged ≥18 years with T2DM, cancer, and exposure to anthracyclines	640 SGLT2i recipients/640 SGLT2i non-recipients	EMPA CANA DAPA	↓ Acute HF exacerbations; ↓ All-cause mortality; Fewer hospitalizations and emergency department visits; ↓ Incidence of AF; ↓ Occurrence of AKI and need for renal replacement therapy;
Henson et al. (2024) [162]	Patients with HF previously treated with anthracyclines	1323 SGLT2i recipients/1323 SGLT2i non-recipients	EMPA CANA DAPA	↓ All-cause mortality;

AF = atrial fibrillation; AKI = acute kidney injury; CANA = canagliflozin; DAPA = dapagliflozin; T2DM = type 2 diabetes mellitus; EMPA = empagliflozin; HF = heart failure; MI = myocardial infarction; SGLT2i = SGLT2 inhibitor; ↓ = decreases; ↑ = increases.

## Abbreviations

ACE-I—angiotensin-converting enzyme inhibitors; AF—atrial fibrillation; AIC—anthracycline-induced cardiotoxicity; AIF—apoptosis-inducing factor; ANT—Adenine Nucleotide Translocator; ARB—angiotensin receptor blockers; BNP = B-type natriuretic peptide; BP = blood pressure; CANA—canagliflozin; CAT—catalase; CHF—congestive heart failure; CKD—chronic kidney disease; CMR—cardiac magnetic resonance; CTRCD—cancer therapy-related cardiac dysfunction; CypD—cyclophilin D; DAPA—dapagliflozin; DLBCL—diffuse large B-cell lymphoma; DOX—Doxorubicin; ECG—electrocardiogram; EMPA—empagliflozin; FV—fluvastatin; GDF-15—growth differentiation factor-15; GLS—global longitudinal strain; GPx—glutathione peroxidase; H_2_O_2_—hydrogen peroxide; HF—heart failure; HFmrEF—HF with mildly reduced ejection fraction; HFpEF—HF with preserved ejection fraction; HFrEF—HF with reduced ejection fraction; hs-cTnT—high-sensitivity cardiac troponin T; hs-CRP—high-sensitivity C-reactive protein; IL-1β—interleukin-1 beta; IL-6—interleukin-6; iPSC-CMs—induced pluripotent stem cell-derived cardiomyocytes; LASr—left atrial reservoir strain; LAV—left atrial volume; LVEF—left ventricular ejection fraction; LVSD—left ventricular systolic dysfunction; MI—myocardial infarction; MTP—myeloperoxidase; mPTPs—mitochondrial permeability transition pores; NADPH—nicotinamide adenine dinucleotide phosphate; NF-κB—nuclear factor kappa-B; NHL—non-Hodgkin’s lymphoma; NOSs—nitric oxide synthases; NPs—natriuretic peptides; NT-proBNP—N-terminal pro-B-type natriuretic peptide; RAS—renin–angiotensin system; RNS—reactive nitrogen species; ROS—reactive oxygen species; SGLT2i—sodium-glucose cotransporter-2 inhibitors; SNPs—single nucleotide polymorphisms; SOD—superoxide dismutase; T2DM—type 2 diabetes mellitus; TNF-α—tumor necrosis factor-alpha; TnI—troponin I; TTE—transthoracic echocardiography; QTc = corrected QT.
